# Can biological structures be natural and sustainable capsules?

**DOI:** 10.3389/fchem.2015.00036

**Published:** 2015-06-10

**Authors:** Bao-Ngoc Pham-Hoang, Hanh Phan-Thi, Yves Waché

**Affiliations:** ^1^UMR PAM Food and Microbial Process, AgroSup Dijon, University of BurgundyDijon, France; ^2^NatencapsDijon, France

**Keywords:** natural capsules, biological structures, actives, fragrances, protection, controlled release

Flavor and fragrance molecules are used in many industrial fields such as food, cosmetics, tissues, pharmacy, agriculture (pheromones) etc. As most actives have a specific target and are fragile molecules, encapsulation processes have been developed for their use. These technologies are efficient to avoid loss of actives, dissemination out of the target and subsequent pollution, and to protect molecules up to their target. Several processes have been developed responding to the numerous situations encountered (e.g., protection against air, temperature, light, pH; masking or revealing sensorial properties of the molecule; release during the process, in the plate, in the mouth, etc.).

However, the general trend for natural products and for processes friendly for the environment has put forward several constraints. According to the various regulations (CFR 1990, CE 1334/2008 etc.), to be natural, a product has to come from plant or animal raw materials with only a physical, enzymatic or microbial process at the exclusion of any chemical step. This definition results from a consensus between jurists, philosophers, industry, consumers, politics etc. It must be noted that this concept of naturality is neither universal nor consensual. In philosophy, although defined by Aristotle in his Book II as one thing that has “within itself a principle of motion and of stationariness,” natural things are not always easy to recognize from their opposite, artifactual things. With the completed and extended definition of artifactual things to what has been done in a project or purpose [discussed by J. Monod in the introduction of “Chance and necessity” (1971)], agriculture and biotechnology products would become mainly artifactual. This concept of naturality is thus quite controversial and regulation may evolve in the future. However, despite the fact that many definitions are hidden behind the word of naturality, this concept has attracted consumers, first in Germanic countries in the 1980s, spreading over Europe, then to North America, and from there to the whole World. The demand for natural product first appeared in food, reached cosmetics recently and is now expanding to other fields. However, to keep the label, natural flavors and fragrances require natural capsules, i.e., capsules coming from natural materials that have not been modified through chemical steps. As a result, the issue of naturality is closely related to the sustainability of the process, i.e., its impact on the environment (release of solvents, carbon impact etc.), which is also getting more and more important.

In the field of encapsulation, among the numerous technologies developed at the lab scale, just few are ready for industrial implementation and it is often difficult to obtain an up-scalable process without any use of toxic solvents or not-natural materials. These technologies are based on various principles often trying to polymerise and coat a suspension of active. One could notice however that the presence of compartments has been a prerequisite to evolution and natural capsules have thus been developed from the apparition of the first cell. Among biological structures, cells are indeed a good protection for actives that have to interact together and with the environment. When the protection needs to be stronger and fewer relations are required between actives and the environment, specific structures are produced like microbial spores. Eventually, specialized structures are able to disseminate actives in the environment like pollen grains. With such nice model systems, humanity has developed biomimetics to synthesize efficient artificial capsules (Cai et al., 2015) but tries also to use already existing natural capsules (Pham-Hoang et al., 2013). Although several works have been carried out on this subject and some products have been developed from years, these natural capsules are difficult to utilize, precisely because of their envelope properties (Table 1). Indeed, the envelope, that makes the active protected inside the cell, is difficult to cross in a controlled way, making loading and controlled release difficult. Industrial encapsulation in these natural structures might also be difficult to carry out in a fully natural and sustainable process. In this opinion article, we propose to discuss the utilization of biological structure as microcapsules for natural and sustainable odorant products.

**Table 1:** **Performances of various biological structures as natural capsules**.

	**Advantages**	**Disadvantages**	**Illustration**
Yeast cells	Simple process High efficiency for hydrophobic molecules Heat protection (up to 250–270°C) Controlled release Homogenous size 5–10 μm depending on the species)	Solvent can improve loading of hydrophilic molecules or cell performance	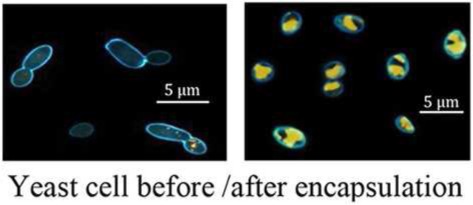
Microbial spores	High potential for protection against heat, UV, and chemicals Homogenous size (0.5–2 μm depending on the species)	Almost impossible to load	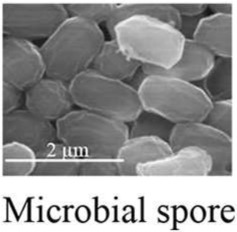
Pollen grain shell	Easy loading Controlled release (e.g., with pH control) Light (up to 80% of UV radiation) and temperature (up to 275°C) protection Homogenous size (3–250 μm depending on the species)	Usually emptied with organic solvents, sometimes in combination with alkali (for DNA) and acid (for polysaccharide extraction) Whitening of shield may be required	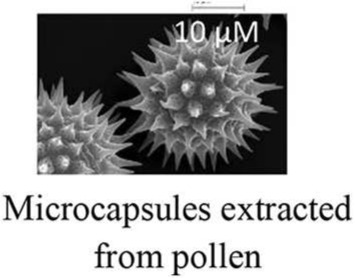
Plant cells	Hydrocarbon solvent free Easy release Without loading step	Difficult to load extracellular compounds Depending on plant species Low efficiency	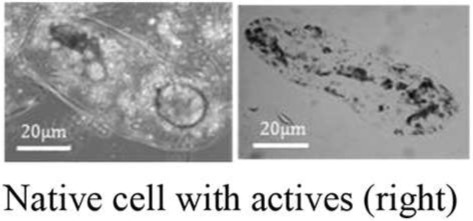

Illustration: Microbial spores: unpublished picture by Nguyen; Microcapsules extracted from pollen picture presented with permission from Diego-Taboada et al. (2014).

## Loading biological structures

The loading of actives inside biological structures is usually a big issue as these structures are good protecting structures and transfer across them is thus difficult to manage. Among biological structures, there is a huge diversity between bacterial spores that are almost non permeable to all kind of actives with a particular role of the highly viscous inner membrane (Loison et al., 2013) and yeast cells in which oil transfers readily up to 70/100 g of dry weight. Therefore, there are several strategies between passive transfer and solvent-based “cleaning” of the shell of the structure as it is done for instance for pollen hollow shell (Diego-Taboada et al., 2014). A treatment of osmoporation has also been proposed to increase the transfer (da Silva Pedrini et al., 2014) showing an interest for the highly hydrosoluble chlorogenic acid (Shi et al., 2007) but not for the less polar curcumin (Paramera et al., 2011).

## Protection provided by biological structures

The protection provided by biological structures is usually very strong and one of the highest available in encapsulation techniques (Table 1). Yeast cells as well as emptied pollen grain and spore shells have the ability to withstand temperatures up to 205 to 275°C (Normand et al., 2005; Diego-Taboada et al., 2014). They provide also a good protection against UV, oxygen, acid, and alkaline stresses. Bacterial spores are possibly the most resistant structure or at least, the structure protecting life in the more drastic conditions of temperature and pressure and many processes aiming at destroying these cells are based on the initiation of germination as the first step of the treatment.

## Release from biological structures

The speed of release can be governed by the kinetic of transfer across the capsule envelope or by the kinetic of degradation/dissolution of the capsule. Plant spores can for instance be enzymatically degraded resulting in a slow release (Lorch et al., 2009). The importance of pH has also been shown with 100% release in a few min at neutral pH but only 10% at acidic pH (Diego-Taboada et al., 2014). In yeast cells, release depends on the presence of water which can be involved in the transfer as already observed with several materials (Hantelys et al., 2012) but also on the protein structure. Dry cells can have an impermeable protein layer at the cell wall surface (Normand et al., 2005). Transfer can also depend on the presence of oil in the outer medium. Bacteria have also been used for surface display and encapsulation in the cell wall. In this case, the outermembrane of cells was only used as an adhesion support matrix and controlled release was obtained through the coating of the negatively-charged bacterial surface by a cationic urea-formaldehyde resin (Zhang et al., 2015). This system is of course incompatible with natural encapsulation but cell-coating through natural polymers of polyelectrolytes like β-lactoglobulin and alginate (Nguyen et al., in press) is also possible.

## In conclusion, are biological structures usable as natural sustainable products?

Biological structures such as pollen, cells or spores possess very efficient protective properties especially against heat stress but also against light or oxygen. Their drawbacks are related with this property as it may be very difficult to load these structures. Several treatments can be used, aiming at increasing permeability, emptying the structure or cleaning it to keep only one protective material. Several chemical or solvent-based treatments together with heat-treatments can be used, decreasing thus the sustainability and the natural essence of the capsule especially when a succession of treatments must be used to extract lipids (organic solvents), genetic materials (alkali), and polysaccharides (acids). However, some enzymatic treatments can also be used and many hydrophobic compounds are readily loaded from the outside medium and concentrated inside cell compartments showing thus the right properties for a sustainable natural process.

Among the various biological structures presented, yeast-cells are the most advanced capsules with already several products on the market. It is performed with a simple process that can be scaled up for industrial purpose. Up to now, this technology was developed and patented by many companies for use in cosmetics, textiles, drug delivery, and especially in food flavors, etc. (detailed in Pham-Hoang et al., 2013). The yeast capsules are often in powder form that can favor the easy handling, usage, and conservation (Baines and Knights, 2009). In term of production cost, the price of yeast capsules is comparable with the one of other materials due to the existence of a highly optimized market of active and inactive yeasts and to the possibility to load simply and up to 70–80% of actives. Pollen shields have been the subject of many studies for drug delivery and are at the development stage but high protection, important loading (up to 80%), monodispersed size, low cost (for drug applications), and increased bioavailability of drugs give them a high potential of development. However, to obtain a natural label, the key step is to empty shields before loading (Diego-Taboada et al., 2014).

In conclusion, biological structures are really efficient encapsulation structures that are mainly used with the help of solvent-based treatments but that can also be used as natural and sustainable capsules in many cases. Their transformation from biology to technology materials requires a good knowledge of cell biology as well as of transfers and encapsulation and an acceptation of huge naturality and sustainability constraints as in many food biotechnology fields.

### Conflict of interest statement

The authors declare that the research was conducted in the absence of any commercial or financial relationships that could be construed as a potential conflict of interest.
